# Molecular Modelling Study of the PPARγ Receptor in Relation to the Mode of Action/Adverse Outcome Pathway Framework for Liver Steatosis

**DOI:** 10.3390/ijms15057651

**Published:** 2014-05-05

**Authors:** Ivanka Tsakovska, Merilin Al Sharif, Petko Alov, Antonia Diukendjieva, Elena Fioravanzo, Mark T. D. Cronin, Ilza Pajeva

**Affiliations:** 1 Institute of Biophysics and Biomedical Engineering—BAS, Acad. G. Bonchev Str., Bl.105, Sofia 1113, Bulgaria; E-Mails: merilin.al@biomed.bas.bg (M.A.S.); petko@biophys.bas.bg (P.A.); antonia.diuk@gmail.com (A.D.); 2 Soluzioni Informatiche srl, Via Ferrari 14, Vicenza 36100, Italy; E-Mail: elena.fioravanzo@s-in.it; 3 School of Pharmacy and Biomolecular Sciences, Liverpool John Moores University, Byrom Street, Liverpool L3 3AF, UK; E-Mail: M.T.Cronin@ljmu.ac.uk

**Keywords:** PPARγ, liver steatosis, mode of action, molecular modelling, pharmacophore

## Abstract

The comprehensive understanding of the precise mode of action and/or adverse outcome pathway (MoA/AOP) of chemicals has become a key step toward the development of a new generation of predictive toxicology tools. One of the challenges of this process is to test the feasibility of the molecular modelling approaches to explore key molecular initiating events (MIE) within the integrated strategy of MoA/AOP characterisation. The description of MoAs leading to toxicity and liver damage has been the focus of much interest. Growing evidence underlines liver PPARγ ligand-dependent activation as a key MIE in the elicitation of liver steatosis. Synthetic PPARγ full agonists are of special concern, since they may trigger a number of adverse effects not observed with partial agonists. In this study, molecular modelling was performed based on the PPARγ complexes with full agonists extracted from the Protein Data Bank. The receptor binding pocket was analysed, and the specific ligand-receptor interactions were identified for the most active ligands. A pharmacophore model was derived, and the most important pharmacophore features were outlined and characterised in relation to their specific role for PPARγ activation. The results are useful for the characterisation of the chemical space of PPARγ full agonists and could facilitate the development of preliminary filtering rules for the effective virtual ligand screening of compounds with PPARγ full agonistic activity.

## 1. Introduction

Modern toxicology concepts aim at building alternative models (*in vitro* and *in silico*) to predict the adverse effects of chemicals. This requires comprehensive understanding of biological pathways starting at the molecular level and their relationship to adverse effects at the organ and higher levels of organism organization [1]. These concepts are based on the adverse outcome pathway (AOP) methodology and lie at the heart of initiatives, such as SEURAT-1 (http://www.seurat-1.eu) and TOX21 (http://www.epa.gov/ncct/Tox21/). The AOP methodology supports the use of a mode of action (MoA) framework involving the description and characterisation of the key cytological and biochemical events that are both measurable and necessary for the observed effect [2].

Within the MoA/AOP framework, the description and characterisation of the toxicological MoAs leading to liver toxicity are of specific interest, since the liver is a major organ affected by toxicity. *In silico* approaches are suitable tools to study the starting events of these MoAs—the molecular initiating events (MIE). Recently, molecular modelling approaches have been proposed for the study of the MIEs involved in liver steatosis [3,4]. They include hepatic PPARγ activation, which has been noted as being one of the probable MIEs leading to non-alcoholic fatty liver disease, including liver steatosis [5,6].

The peroxisome proliferator-activated receptor gamma (PPARγ) is a member of the steroid-thyroid hormone superfamily of ligand-activated transcription factors. It has been well studied in the last decade, because of its important role in glucose and lipid homeostasis. The mechanisms of PPARγ genomic activity (transactivation and transrepression) have been comprehensively studied. The nuclear receptor forms a heterodimer with another nuclear receptor, retinoid X receptor alpha (RXRα), and binds to specific DNA sequences in the promoter regions of target genes. PPARγ binds and responds to diverse endogenic lipid metabolites, including eicosanoids and fatty acids [7]. The activation of PPARγ is induced by specific conformational changes upon ligand binding; these changes release corepressors and allow for the recruitment of coactivators.

**Figure 1.**
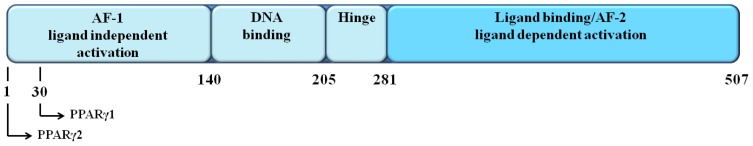
Schematic structure of the functional domains of the PPARγ isoforms (transactivation function domain 1 (AF1) DNA-binding domain (DBD), ligand-binding domain (LBD) and transactivation function domain 2 (AF2)).

PPARγ has two isoforms, PPARγ1 and PPARγ2, the latter possessing an additional thirty amino acids in the *N*-terminal part. It has an overall domain structure typical for nuclear hormone receptors comprised of: an *N*-terminal AF-1 (transactivation function 1) domain that participates in the interaction with cofactors and is responsible for ligand-independent transactivation; a DBD (DNA binding domain) that is highly conserved among nuclear receptors; a highly flexible hinge region, necessary for nuclear localisation and cofactor docking; and a *C*-terminal LBD/AF-2 (ligand binding domain/activation function 2) that participates in ligand-binding, ligand-dependent transactivation, coactivator recruitment and corepressor release (Figure 1).

Synthetic PPARγ ligands are primarily categorised based on their transactivation activity into full and partial agonists [8]. The full agonists, despite their clinical benefit (e.g., as antidiabetic agents [9]), have been associated with adverse side effects, including hepatotoxicity [10,11]. Thus, the prediction of the full agonistic effect of synthetic PPARγ ligands is of specific interest in the field of toxicology.

Significant efforts have been made in understanding the ligand-dependent activation of PPARγ. Studies show that binding to the helix12 (H12) of the receptor is required for full agonist activity [12]. Full agonists that interact with PPARγ generally involve a polar terminal group and a hydrophobic moiety. The polar group forms hydrogen bonds (HBs) with the receptor residues Ser289, His323, His449 and Tyr473. These HBs are responsible for the conformational change of H12 and, subsequently, the activation of PPARγ [13].

In this study, we performed a comprehensive analysis of the PPARγ binding pocket and the interactions of full agonists-PPARγ based on the 3D PPARγ complexes deposited in the Protein Data Bank (http://www.rcsb.org) [14]. A pharmacophore model was developed based on the most active ligands, and it was evaluated on a dataset of full agonists extracted from PDB. The pharmacophore features were evaluated according to their role in PPARγ interactions and the transactivation activity of the full agonists.

## 2. Results and Discussion

### 2.1. Analysis of the PPARγ LBD and Ligand-Receptor Interactions

**Figure 2.**
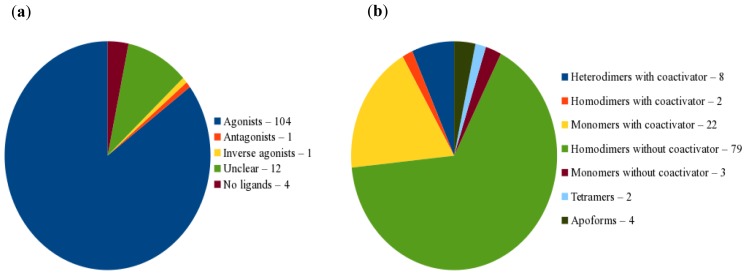
Distribution of the structures according to the type of: (**a**) the bound ligands; (**b**) the receptors.

The PPARγ LBD and the ligand interactions were analysed based on the agonist complexes extracted from PDB and subsequently 3D-protonated at appropriate physiological conditions to assign the correct ionisation state and positions of the missing H-atoms. The complexes differed in the type of the bound ligands (Figure 2a) and the receptors (Figure 2b). Some of the complexes had two ligands simultaneously occupying the LBD [15–17].

The protein structures of the complexes were next superposed on the X-ray structure of the PPARγ-rosiglitazone complex (PDB ligand ID BRL; complex ID 1FM6, [18]). This complex was selected as an appropriate template in the subsequent modelling steps, because of the following reasons: (i) the complex represents a physiologically relevant heterodimer of the human RXRα and PPARγ LBDs, respectively, bound with 9-cis retinoic acid and rosiglitazone and co-activator peptides; (ii) rosiglitazone is one of the most active agonists among the PPARγ ligands (Table S1), thus providing a relevant structure for the purposes of pharmacophore modelling; (iii) the LBD of the complex consists of 272 residues (from Pro206 to Tyr477), thus fully covering the main structural elements of the domain; (iv) compared to other complexes of PPARγ with rosiglitazone (4EMA, 3DZY, 2PRG, 3CS8, [19–22]) available in PDB, that selected has the lowest resolution of 2.1 Å (the complex with PDB ID 1ZGY [23] has been resolved at 1.80 Å; however, it is not crystallised with RXRα and, thus, does not reflect physiological conditions). Due to this, the 1FM6 complex appears to be the most appropriate template, due to its physiologically relevant form and low resolution.

The complexes were superposed on the C-alpha atoms of both X and D-chains of the 1FM6 complex, and the root-mean-square deviation (RMSD) values were recorded. In general, the superposition on the D-chain produced better RMSDs; thus, the superposition on the D-chain of the 1FM6 complex was selected to produce the overlay of all bioactive conformations of the PPARγ full agonists.

**Figure 3.**
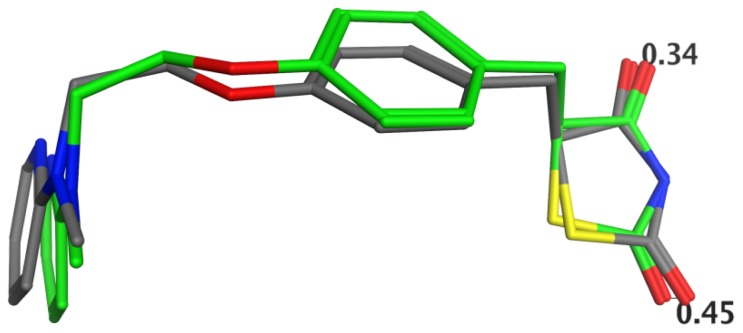
Superposed structures of rosiglitazone: the X-ray structure (PDB ID 1FM6) is shown in the atom type colour and the carbon atoms of the structure optimised by the MMFF94s force field are coloured in green. The structures are superposed on the heteroatoms, and the distances between the oxygen atoms in the thiazolidine ring are in Å.

Considering the fact that the X-ray structure may represent a “tensed” conformation of the ligand due to the crystal packing forces, we compared the structure of rosiglitazone as extracted from the 1FM6 complex and the one that has been relaxed using the MMFF94s force field. The X-ray and relaxed structures were very close (RMSD 0.388 Å upon superposition on all heavy atoms and 0.377 Å on the heteroatoms only). Figure 3 illustrates the structures superimposed on the heteroatoms. The distances between the oxygen atoms were 0.34 and 0.45 Å, and the nitrogen atoms in the thiazolidine rings were fully overlaid. As shown below, these atoms are involved in the specific interactions of rosiglitazone with PPARγ. The small differences in the space location of the most important atoms imply that the X-ray ligand conformation may not be strongly influenced by the crystallisation. To support this suggestion further, we superposed the heavy atoms of the rosiglitazone structure extracted from all available complexes. The RMSD values in the interval 0.18–0.58 Å (template: rosiglitazone structure from 1FM6 complex, D chain) were recorded, indicating that the variations in rosiglitazone conformations between the complexes were small (Table S1). This is in agreement with previous studies on the optimisation of X-ray complexes of another nuclear receptor (human estrogen receptor α) at different levels of protein flexibility and, so, points to the fact that the ligand X-ray structures represent a stable bioactive conformation [24].

**Figure 4.**
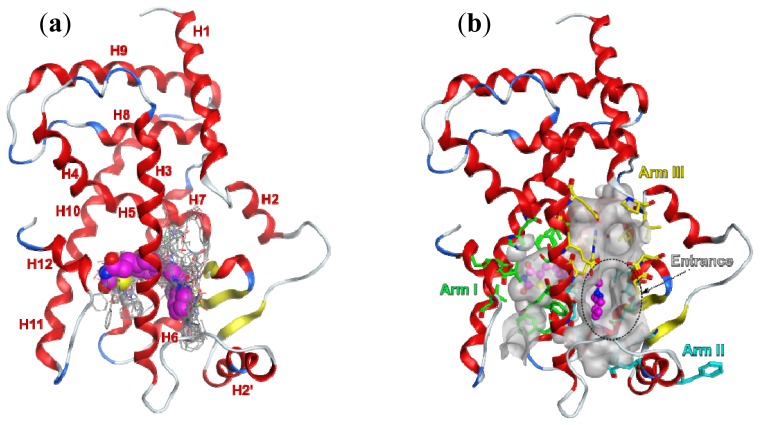
(**a**) PPARγ and 58 agonists superposed on the template complex PDB ID 1FM6 with rosiglitazone (in space-filled rendering and C-atoms coloured in magenta); the other ligands are rendered in lines and coloured according to atom type; (**b**) surface map of the binding site (in constant grey colouring) of all agonists and rosiglitazone (in magenta); the coloured residues outline the corresponding arms within the binding site: Arm I, green; Arm II, cyan; Arm III, yellow; the entrance to the pocket (outlined with a black dotted line) is located between the arms; the protein backbone is rendered in ribbon and coloured according to the secondary structure: helix, red; strand, yellow; turn, blue; loop, white; H1–H12 assign the numbers of the helices in the PPARγ LBD structure.

Figure 4a illustrates the PPARγ LBD and 58 full and partial agonists superposed on the template complex 1FM6 with rosiglitazone. Figure 4b shows the template structure with the binding pocket outlined by its surface (within 4.5 Å of the ligand atoms). The binding pocket is large (~1300 Å^3^, [21]) and can accommodate more than one ligand in more than one binding mode. It has a complex form, as seen from the three main directions (Arm I, II and III) occupied by the bound ligands. The entrance to the pocket does not coincide with either of the arms: it is in the direction of the anchor point of the arms and is located between H3 and the strands. In the binding pocket, the polar parts of the ligands are directed to H12 in Arm I; the helix proved to play a key role for the binding of coactivators.

**Figure 5.**
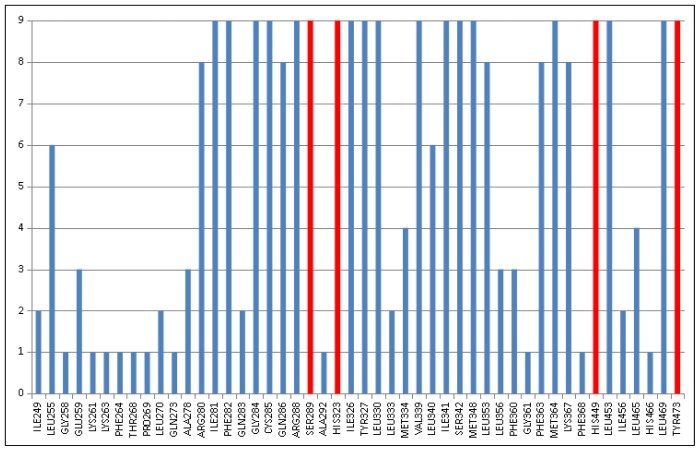
The number of occurrences of the amino acids involved in the binding pockets of the nine most active agonists (according to the EC_50_ values in Table S1); in red are the amino acids that were identified to form hydrogen bonds (HBs) with the most active agonists.

Analysis of the binding pocket of the nine most active agonists described the amino acids forming the receptor-binding pocket (Figure 5). Nineteen out of 48 residues are involved in the binding sites of all agonists, and Ser289, His323, His449 and Tyr473 (shown in red) have been detected to form hydrogen bonds in the analysis of the ligand-receptor interactions of the most active agonists. At the same time, sixteen residues have been detected in one or two complexes, indicating some flexibility of the binding site depending on the bound ligand.

**Figure 6.**
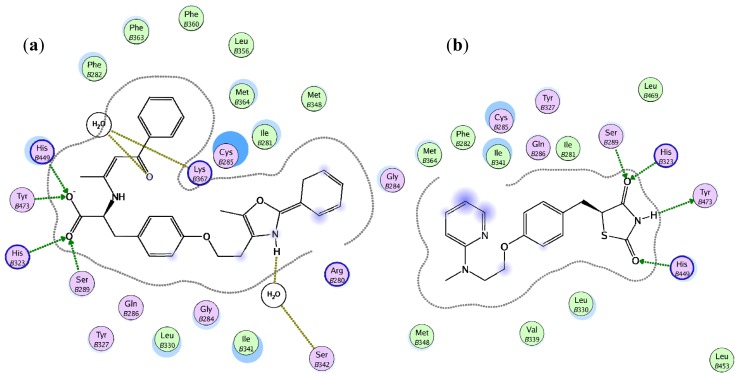
Ligand-interaction diagrams of (**a**) rosiglitazone and (**b**) GW409544 within the binding pocket of PPARγ.

Figure 6 illustrates the ligand-protein interactions of rosiglitazone in the PPARγ complex 1FM6 and GW409544 (PDB ligand ID 544) in complex 1K74 [25]. Ser289, His323, His449 and Tyr473 were the key receptor residues involved in HB interactions with the ligands.

**Figure 7.**
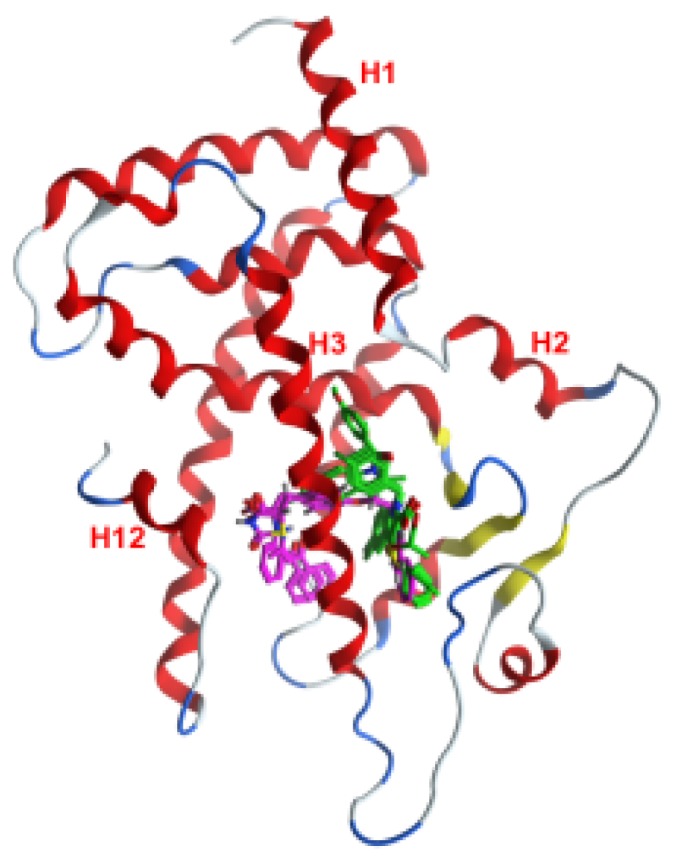
Binding poses of three full agonists (BRL (rosiglitazone), 544 (GW409544) 570 (farglitazar); in magenta) and three partial agonists (MRL24, SR145, SR147; in green) within the PPARγ binding pocket (template complex 1FM6).

Inspection of the binding pocket of all agonists confirms the observations that partial agonists occupy different regions, suggesting different modes of binding from H12 stabilisation for activation of PPARγ by the partial agonists (Figure 7) [12].

### 2.2. Pharmacophore Development

**Figure 8.**
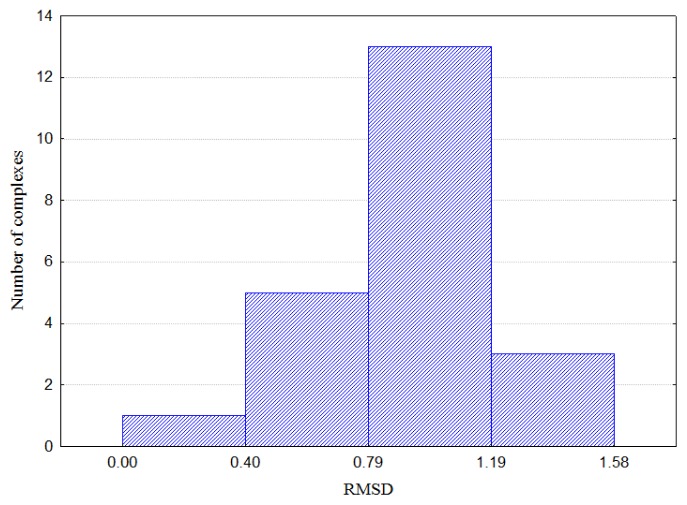
Histogram of the RMSD values (*X*-axis) of superposed PPARγ-full agonist complexes (*Y*-axis).

Prior to pharmacophore development, the full agonists’ complexes were superposed on the template structure of 1FM6. The RMSD values of the superposed structures are reported in Table S1, and Figure 8 illustrates the histogram of the RMSD values. The RMSD values over the superposed structures vary from 0.44 to 1.58 Å. The largest number of structures falls into the interval 0.8–1.2 Å. A closer investigation of the deviations of the protein chains showed that they relate mostly to the loop between H2′ and H3 (Figure 4a), while the helices that form the binding site appear well superposed. The flexibility of the H2′-H3 loop suggests that it might play a role in the accommodation of ligands of different form and size and, hence, preserving the positions of the helices in the PPARγ binding pocket. Thus, the PPARγ binding site remains relatively stable upon binding of different ligands, and the superposed ligands can represent a reliable alignment for pharmacophore generation.

**Figure 9.**
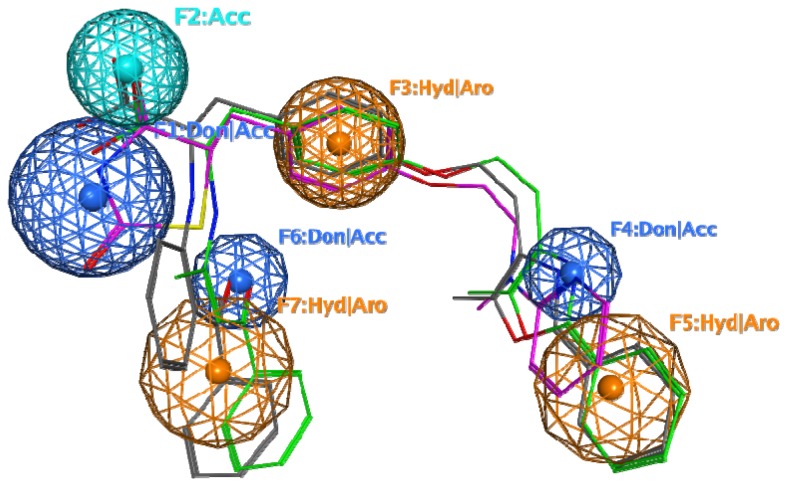
Pharmacophore model of PPARγ full agonists (rosiglitazone, carbon atoms in magenta; compound 544, carbon atoms in green; compound 570, carbon atoms in grey).

The pharmacophore model was built based on the three most active agonists (Figure 9). It outlines seven important pharmacophore features that were observed in the most active agonists: four polar atoms and functional groups capable of performing HB and ionic interactions (F1, F2, F4 and F6) and three hydrophobic and aromatic structural elements (F3, F5 and F7). The hydrophobic/aromatic features stabilize the positions of the hydrophilic ones; the terminal F5 and F7 features are directed inside the pocket Arms I and II and contribute additionally to the stabilisation of the ligand pose into the pocket, ensuring the optimal position of the F1 and F2 features. Similar functions can be assigned to the F4 and F6 features interacting with the protein residues, either directly or through water molecules. The pharmacophore features and their possible role in the interaction within the binding pocket are summarised in Table 1.

It should be noted that the seven-feature pharmacophore model is rather restrictive, since it is based on the most active agonists. Thus, the pharmacophore model was evaluated further among 21 full agonists from PDB, selected based on reliable data about their full agonistic activity and experimental transactivation EC_50_ values available in the source papers. The search was performed by superposition of the agonist structures on the pharmacophore model and estimation of the correspondence between pharmacophore points and their respective substructures. The results are reported in Table 2. Most of the compounds comprise 4–5 pharmacophore features. All of them contain either F1 or F2 or both simultaneously. The other pharmacophore features stabilise the position of the ligand in the pocket: in Arm II (by F4 and/or F5) or in Arm I (by F6 or/and F7). Features F1 or/and F2 and F3 could be outlined as mandatory for full agonism; at least one of the other features (F4–F7) is necessary for the full agonism. The most active agonists contain the highest number of pharmacophore features, whilst some of them are missing from the less active agonists.

In order to investigate in details how H12 is influenced by the ligand interactions, an analysis of the HB interactions between the ligands and the protein, as well as the contacts between amino acids of H12 and its vicinity and other amino acids in the PPARγ LBD was performed for the 21 complexes with full agonists. The apo-form (1PRG, [21]) was also included in the analysis for comparison. The results are summarised in Table S2. A number of ligands interact directly with H12 through HBs (e.g., 544, 570, BRL, ZAA), thus fitting to the F1 feature. For others (e.g., M7R, M7S, S44, J53) no interactions are identified with H12; instead, they interact with H3 and/or H5 fitting in this way with the F2 feature. At the same time, inspection of the protein contacts between the amino acids of H12 and its vicinity to other amino acids in the LBD reveals unique HBs that take place in complexes only and are not observed in the apo-forms. These new contacts connect H12 to H3, H4, and H5, thus stabilising its active position (e.g., Ile472 (H12) with Lys319 (H4), Lys474 (after H12) with Lys319 (H4), Tyr477 (after H12) with Glu324 (H5), Hys466 (between H10/11 and H12) with Gln 286 (H3); Table S2, highlighted lines). In the most active agonists, the HB contacts between H12 and H4 are recorded; in those that miss F1, HB contacts of H12 to H3 prevail. Obviously, such interactions facilitated by the ligand binding contribute to the stabilisation of the active position of H12, necessary for coactivator recruitment.

## 3. Experimental Section

MOE software was used to prepare structures (3D-protonated), to superpose and calculate the RMSD values of the extracted complexes and to develop the pharmacophore model of the PPAR full agonists [43].

### 3.1. PPARγ Structural and Activity Data

One hundred twenty available complexes of the human PPARγ receptor were extracted from PDB (last access: 15 February 2014). The experimental PPARγ activity data for the ligands identified in the complexes were collected from PDB and ChEMBL databases [44]. The data include binding affinity and transcriptional activity values (K_i_, K_d_, IC_50_, EC_50_) as obtained in competition binding assays, the scintillation proximity assay and transcriptional activation assays (Table S1).

### 3.2. Preparation of the Protein Structures (Protonate 3D)

In order to prepare the initial structures of the receptor, namely to assign the correct ionisation states and to position hydrogen atoms in the X-ray PPARγ protein structures, the MOE tool “Protonate 3D” was used. The addition of hydrogen atoms by this application is related to the determination of: the rotamers of –SH, –OH, –CH_3_ and –NH_3_ groups in Cys, Ser, Tyr, Thr, Met, Lys; the ionisation states of acids and bases in Arg, Asp, Glu, Lys, His; the tautomers of imidazoles (His) and carboxylic acids (Asp, Glu); the protonation state of metal ligand atoms in Cys, His, Asp, Glu, *etc*.; the ionisation state of metals; and the element identities in His and the terminal amides (Asn, Gln). In the application, the generalized Born/volume integral electrostatics model was used for the optimization of the titration free energies of all titratable groups. The physiologically relevant conditions were set (temperature: 310 K; pH = 7.4; ion concentration: 0.152 mol/L).

### 3.3. Analysis of the Ligand-Receptor Interactions

The MOE tool “Ligand Interactions” was applied to perform the analysis of the ligand-receptor interactions in the PPARγ complexes. The application identifies a number of interactions (hydrogen bonds, salt bridges, hydrophobic interactions, cation-π, sulphur-lone pair, halogen bonds and solvent exposure) between the ligand and the receptor-interacting entities as HB residues, close, but non-bonded residues (approaching the ligand, but not having any strong interactions, *i.e.*, HBs), solvent molecules and ions. The HB interactions between pairs of heavy atoms from the ligand and the receptor are identified according to probability criteria derived from a large training set. The HB scores are expressed as percentages, and the HB directionality is noted. In the study, the default were applied.

### 3.4. Superposition of the Complexes

The LBDs of all extracted PDB complexes of PPARγ agonists were superposed on a template structure by the C-alpha atoms, and the RMSD values were recorded using the “Protein superpose” tool in MOE.

### 3.5. Pharmacophore Development

The pharmacophore was developed using the “Pharmacophore Query Editor” tool in MOE. The application refers to the generation of a pharmacophore hypothesis for the binding interactions in a particular active site. In MOE, the computerised representation of a hypothesised pharmacophore is called a pharmacophore query. An MOE pharmacophore query is a set of query features that are typically created from ligand annotation points.

Annotation points can be broadly divided into three categories, atom, projected and centroid, the latter including bioisosteres: (i) atom annotations are located directly on an atom of a molecule and typically indicate a function related to protein-ligand binding (these include the H-bond donor (Don), the H-bond acceptor (Acc), cation (Cat), anion (Ani), metal ligator (ML) and hydrophobic atom (HydA); (ii) projected annotations are located along implicit lone pair or implicit hydrogen directions and are used to annotate the location of possible partners for hydrogen bond or metal ligation or possible R-group atom locations. These include: projected donor (Don2), projected acceptor (Acc2), projected metal ligator (ML2) and ring projection (PiN); and (iii) centroid annotations are located at the geometric centre of a subset of the atoms of a molecule: aromatic (Aro), pi-ring (PiR) and hydrophobic (Hyd).

The annotation points on a ligand are the potential locations of the features that will constitute the pharmacophore query and are automatically detected in MOE. Annotation points relevant to the pharmacophore are converted into query features with the addition of an extra parameter: a non-zero radius that encodes the permissible variation in the pharmacophore query’s geometry.

## 4. Conclusions

In summary, a comprehensive analysis was performed on the available PDB complexes of PPARγ with full agonists. A pharmacophore model was derived demonstrating the importance of hydrogen bonding and hydrophobic features for full agonistic activity. The pharmacophore features were evaluated according to their role in the interaction with PPARγ H12 and the transactivation activity of the full agonists. It is envisaged that the pharmacophore model developed will be used for the *in silico* screening of agonists of hepatic PPARγ that can function as steatogenic inducer molecules.

## Supplementary Information



## Figures and Tables

**Table 1.** Description of the pharmacophore features in the pharmacophore model of the full PPARγ agonists. Don, donor; Acc, acceptor; Hyd, hydrophobic; Aro, aromatic.

Pharmacophore feature	Location	Interactions
F1: Don/Acc	Arm I	Participates in HB interactions (donor and acceptor) with residues His449 (H11) and Tyr473 (H12); responsible for the direct interaction with H12 and stabilises its active position
F2: Acc	Arm I	Participates in HB interactions (acceptor) with Ser289 (H3), His323(H5), Tyr 327 (H5); responsible for the stabilization of H12 in an active position
F3: Hyd/Aro	Arm I	Fits to the hydrophobic environment; stabilises the positions of F1 and F2 features
F4: Don/Acc	Arm II	Can participate in HB interactions directly or mediated by water molecules with Ser342 (H5), Cys285 (H3) and Arg 288 (H3); stabilises the pose of the ligand into the pocket
F5: Hyd/Aro	Arm II	Fits to the hydrophobic environment; stabilises the pose of the ligand into the pocket
F6: Don/Acc	Arm I	Can participate in HB interactions mediated by water; stabilises the pose of the ligand into the pocket
F7: Hyd/Aro	Arm I	Fits to the hydrophobic environment; stabilises the pose of the ligand into the pocket

**Table 2.** Evaluation of the pharmacophore model on a dataset of full agonists: F1–F7, pharmacophore features; +/−, the presence or absence of the particular pharmacophore feature in the particular chemical structure; EC_50_, transactivation activity; the complexes are ordered according to their EC_50_ values (the lowest value considered when the interval data are reported).

Complex PDB ID, [Ref.]	Ligand PDB ID	Pharmacophore features							EC_50_ (nM)

		F1	F2	F3	F4	F5	F6	F7	
1K74 [25]	544	+	+	+	+	+	+	+	0.2–2.7
1FM9 [18]	570	+	+	+	+	+	+	+	0.339–6
1FM6 [18]	BRL	+	+	+	+	+	−	−	2.4–2880
3AN4 [26]	M7R	−	+	+	+	+	−	−	3.6
3BC5 [27]	ZAA	+	−	+	+	+	+	−	4
3IA6 [28]	UNT	+	+	+	+	+	−	−	13
1I7I [29]	AZ2	+	+	+	−	−	−	+	13–3528
3G9E [30]	RO7	+	+	+	+	+	−	−	21
3AN3 [26]	M7S	−	+	+	+	+	−	−	22
2ZNO [31]	S44	−	+	+	+	+	−	−	41–70
3GBK [32]	2PQ	+	+	+	+	+	−	−	50
3VJI [33]	J53	−	+	+	−	+	−	−	58
2F4B [34]	EHA	+	−	+	−	+	−	−	70
2Q8S [35]	L92	+	+	+	+	+	−	−	140
1KNU [36]	YPA	+	+	+	+	+	−	+	170
3FEJ [37]	CTM	+	+	+	−	+	−	+	210
2HWR [38]	DRD	+	+	+	−	+	−	−	210
2ATH [39]	3EA	+	+	+	−	+	−	−	230
2XKW [40]	P1B	+	+	+	+	+	−	−	280
1NYX [41]	DRF	+	+	+	−	+	−	−	570–600
2GTK [42]	208	+	+	+	+	+	−	+	760
