# Supplementary material for: Molecular Modelling Study of the PPARγ Receptor in Relation to the Mode of Action/Adverse Outcome Pathway Framework for Liver Steatosis
Source: Int J Mol Sci. 2014 May 5;15(5):7651–66. doi: 10.3390/ijms15057651 (PMC4057697; doi:10.3390/ijms15057651)
Supplement: Supplementary file 1 [file ijms-15-07651-s001.pdf]

Supplementary Information

Table S1. Information about PPARγ-full agonist complexes extracted from PDB: complex ID, ligand (agonist) ID, activity data of the PPARγ agonists extracted from PDB and CheMBL databases; RMSD values are recorded after the superposition of all extracted agonist-PPARγ complexes on the template structure from the 1FM6 complex.

Complex PDB ID	Ligand PDB ID	Biological activity				RMSD
		EC_{50} (nM)	K_i (nM)	K_d (nM)	IC_{50} (nM)	
1K74	544	0.2–2.7	1			1.07
1FM9	570	0.339–6	1–1.1	25–217		0.44
1FM6	BRL	2.4–2,880	8–440	30–450	120–4980	0 (template)
3AN4	M7R	3.6				1.20
3BC5	ZAA	4		5		1.51
3IA6	UNT	13			3	0.85
1I7I	AZ2	13–3528	18–200	200–350		1.01
3G9E	RO7	21		19		0.63
3AN3	M7S	22				1.06
2ZNO	S44	41–70				1.15
3GBK	2PQ	50				1.03
3VJI	J53	58				1.04
2F4B	EHA	70			50	1.01
2Q8S	L92	140	140			0.85
1KNU	YPA	170			170	1.58
3FEJ	CTM	210	740	740		0.62
2HWR	DRD	210				0.79
2ATH	3EA	230		152–152.05		0.90
2XKW	P1B	280				1.03
1NYX	DRF	570–600	90	92		1.15
2GTK	208	760		250		0.67

Table S2. Analysis of the HB contacts between amino acids in H12 and in other helices and between full agonists and the receptor in the LBD of the 21 PPARγ complexes extracted from PDB; 1PRG, apo-form.

Complex PDB ID	Ligand PDB ID	HBs between amino acids in the vicinity of H12				HBs between ligand and receptor		
		AA1		AA2		PHF	AA	SE
		AA	SE	AA	SE			
1K74	544	Glu460	H10/11_H12	Arg357	H6_H7	F1	Tyr473	H12
		Ile472	H12	Lys319	H4	F1	His449	H10/11
		Lys474	H12_	Lys319	H4	F2	His323	H5
		Tyr477	H12_	Glu324	H5	F2	Ser289	H3
1FM9	570	Glu460	H10/11_H12	Arg357	H6_H7	F1	Tyr473	H12
		Ile472	H12	Lys319	H4	F1	His449	H10/11
		Lys474	H12_	Lys319	H4	F2	His323	H5
		Tyr477	H12_	Glu324	H5	F2	Ser289	H3
		His449	H10/11	Lys367	H7			
		Lys367	H7	Phe363	loop in H7			
1FM6	BRL	Glu460	H10/11_H12	Arg357	H6_H7	F1	His449	H10/11
		Arg357	H6_H7	Glu276	H2'_H3	F2	His323	H5
		Ile472	H12	Lys319	H4	F2	Ser289	H3
		Lys474	H12_	Lys319	H4			
		Tyr477	H12_	Glu324	H5			
3AN4	M7R	Glu460	H10/11_H12	Arg357	H6_H7	F2	His323	H5
		Arg357	H6_H7	Glu276	H2'_H3	F2	Tyr327	H5
		Ser464	H10/11_H12	Gln286	H3	F4	Cys285	H3
		Leu465	H10/11_H12	Gln286	H3			
		His466	H10/11_H12	Gln286	H3			
		Ile472	H12	Lys319	H4			
		Lys474	H12_	Lys319	H4			
		His449	H10/11	Lys367	H7			
		Lys367	H7	Phe363	loop in H7			
3BC5	ZAA	Ser464	H10/11_H12	Gln283	H3	F1	Tyr473	H12
		His466	H10/11_H12	Gln286	H3	F1	His449	H10/11
		Asp475	H12	Lys319	H4			
		His449	H10/11	Lys367	H7			
		Lys367	H7	Phe363	turn in H7			

Table S2. *Cont.*

Complex PDB ID	Ligand PDB ID	HBs between amino acids in the vicinity of H12				HBs between ligand and receptor		
		AA1		AA2		PHF	AA	SE
		AA	SE	AA	SE			
3IA6	UNT	Glu460	H10/11_H12	Arg357	H6_H7	F1	Tyr473	H12_
		Arg357	H6_H7	Glu276	H2'_H3	F1	His449	H10/11
		His466	H10/11_H12	Gln286	H3	F2	His323	H5
		Ile472	H12	Lys319	H4	F2	Ser289	H3
		His449	H10/11	Lys367	H7			
		Lys367	H7	Phe363	loop in H7			
1I7I	AZ2	His466	H10/11_H12	Gln286	H3	F1	Tyr473	H12
		Gln470	H12	Lys474	H12_	F1	His449	H10/11
		Ile472	H12	Lys319	H4	F2	His323	H5
		Lys474	H12_	Lys319	H4	F2	Ser289	H3
		His449	H10/11	Lys367	H7			
		Lys367	H7	Phe363	loop in H7			
3G9E	RO7	Glu460	H10/11_H12	Arg357	H6_H7	F1	Tyr473	H12
		Arg357	H6_H7	Lys358	H6_H7	F1	His449	H10/11
		Arg357	H6_H7	Glu276	H2'_H3	F2	His323	H5
		Met463	H10/11_H12	Lys275	H2'_H3	F2	Ser289	H3
		His466	H10/11_H12	Gln286	H3			
		Ile472	H12	Lys319	H4			
		Lys474	H12_	Lys319	H4			
		His449	H10/11	Lys367	H7			
		Lys367	H7	Phe363	H7			
		Arg397	H8_H9	Glu324	H5			
		Asp396	H8_H9	Arg443	H10/11			
3AN3	M7S	Glu460	H10/11_H12	Arg357	H6_H7	F2	Tyr327	H5
		Ser464	H10/11_H12	Gln286	H3	F4	Cys285	H3
		Leu465	H10/11_H12	Gln286	H3	F4	Ser342	H5_H6
		His466	H10/11_H12	Gln286	H3			
		Ile472	H12	Lys319	H4			
		Lys474	H12_	Lys319	H4			
		Leu476	H12_	Tyr320	H4			
		His449	H10/11	Lys367	H7			
		Lys367	H7	Phe363	loop in H7			
2ZNO	S44	Glu460	H10/11_H12	Thr459	H10/11	F4	Cys285	H3
		Arg357	H6_H7	Glu276	H2'_H3			
		Ile472	H12	Lys319	H4			
		Glu471	H12	Lys319	H4			
		Lys474	H12_	Lys319	H4			
		His449	H10/11	Lys367	H7			
		Lys367	H7	Phe363	turn in H7			

Table S2. *Cont.*

Complex PDB ID	Ligand PDB ID	HBs between amino acids in the vicinity of H12				HBs between ligand and receptor		
		AA1		AA2		PHF	AA	SE
		AA	SE	AA	SE			
3GBK	2PQ	Glu460	H10/11_H12	Arg357	H6_H7	F1	Tyr473	H12
		Arg357	H6_H7	Glu276	H2'_H3	F1	His449	H10/11
		His466	H10/11_H12	Gln286	H3	F2	His323	H5
		Ile472	H12	Lys319	H4	F2	Ser289	H3
		Tyr477	H12_	Glu324	H5			
		Arg397	H8_H9	Glu324	H5			
		Asp396	H8_H9	Arg443	H10/11			
		His449	H10/11	Lys367	H7			
		Lys367	H7	Phe363	H7			
3VJI	J53	Glu460	H10/11_H12	Arg357	H6_H7	F2	Tyr327	H5
		Ser464	H10/11_H12	Gln286	H3	F4	Cys285	H3
		Leu465	H10/11_H12	Gln286	H3			
		Ile472	H12	Lys319	H4			
		Lys474	H12_	Lys319	H4			
		His449	H10/11	Lys367	H7			
		Lys367	H7	Phe363	loop in H7			
		Arg397	H8_H9	Glu324	H5			
		Arg443	H10/11	Glu324	H5			
2F4B	EHA	Glu460	H10/11_H12	Arg357	H6_H7	F1	Tyr473	H12
		Glu460	H10/11_H12	Thr459	H10/11	F1	His449	H10/11
		Arg357	H6_H7	Glu276	H2'_H3			
		Ile472	H12	Lys319	H4			
		Lys474	H12_	Lys319	H4			
		Tyr477	H12_	Glu324	H5			
		Arg397	H8_H9	Glu324	H5			
		Asp396	H8_H9	Arg443	H10/11			
		His449	H10/11	Lys367	H7			
		Lys367	H7	Phe363	turn in H7			
2Q8S	L92	Glu460	H10/11_H12	Arg357	H6_H7	F1	Tyr473	H12
		Ser464	H10/11_H12	Gln283	H3	F2	His323	H5
		Ile472	H12	Lys319	H4	F4	Tyr327	H5
		Glu471	H12	Lys319	H4			
		His449	H10/11	Lys367	H7			
		Lys367	H7	Phe363	turn in H7			
		Arg397	H8_H9	Glu324	H5			

Table S2. *Cont.*

Complex PDB ID	Ligand PDB ID	HBs between amino acids in the vicinity of H12				HBs between ligand and receptor		
		AA1		AA2		PHF	AA	SE
		AA	SE	AA	SE			
1KNU	YPA	Glu460	H10/11_H12	Arg357	H6_H7	F1	Tyr473	H12
		Arg357	H6_H7	Glu276	H2'_H3	F1	His449	H10/11
		Met463	H10/11_H12	Gln283	H3	F2	His323	H5
		Leu465	H10/11_H12	Gln286	H3	F2	Ser289	H3
		His466	H10/11_H12	Gln286	H3			
		Asp475	H12_	Lys319	H4			
		Ile472	H12	Lys319	H4			
		His449	H10/11	Lys367	H7			
		Lys367	H7	Phe363	loop in H7			
		Arg397	H8_H9	Glu324	H5			
3FEJ	CTM	Glu460	H10/11_H12	Arg357	H6_H7	F1	Tyr473	H12
		Asp462	H10/11_H12	Lys275	H2'_H3	F1	His449	H10/11
		Arg357	H6_H7	Glu276	H2'_H3	F2	His323	H5
		His466	H10/11_H12	Gln286	H3	F2	Ser289	H3
		Ile472	H12	Lys319	H4	F4	Arg288	H3
		Lys474	H12_	Lys319	H4			
		His449	H10/11	Lys367	H7			
		Lys367	H7	Phe363	loop in H7			
		Arg397	H8_H9	Glu324	H5			
		Asp396	H8_H9	Arg443	H10/11			
2HWR	DRD	Glu460	H10/11_H12	Arg357	H6_H7	F2	His323	H5
		Arg357	H6_H7	Glu276	H2'_H3	F2	Ser289	H3
		His466	H10/11_H12	Gln286	H3			
		Ile472	H12	Lys319	H4			
		Lys474	H12_	Lys319	H4			
		Arg397	H8_H9	Glu324	H5			
		Asp396	H8_H9	Arg443	H10/11			
2ATH	3EA	Thr459	H10/11_H12	Val455	H10/11	F1	Tyr473	H12_
		Glu460	H10/11_H12	Arg357	H6_H7			
		Arg357	H6_H7	Glu276	H2'_H3			
		Asp462	H10/11_H12	Gln286	H3			
		His466	H10/11_H12	Phe287	H3			
		Lys474	H12_	Tyr320	H4			
		His449	H10/11	Lys367	H7			
		Arg397	H8_H9	Glu324	H5			
		Asp396	H8_H9	Arg443	H10/11			

Table S2. *Cont.*

Complex PDB ID	Ligand PDB ID	HBs between amino acids in the vicinity of H12				HBs between ligand and receptor		
		AA1		AA2		PHF	AA	SE
		AA	SE	AA	SE			
2XKW	P1B	Glu460	H10/11_H12	Arg357	H6_H7			
		Arg357	H6_H7	Glu276	H2'_H3			
		Ser464	H10/11_H12	Gln286	H3			
		His466	H10/11_H12	Gln286	H3			
		Ile472	H12	Lys319	H4			
		Lys474	H12_	Lys319	H4			
		Leu476	H12_	Tyr320	H4			
		His449	H10/11	Lys367	H7			
		Lys367	H7	Phe363	loop in H7			
		Arg397	H8_H9	Glu324	H5			
		Asp396	H8_H9	Arg443	H10/11			
		Arg443	H10/11	Glu324	H5			
1NYX	DRF	Glu460	H10/11_H12	Arg357	H6_H7	F1	Tyr473	H12_
		Ser464	H10/11_H12	Gln286	H3	F2	His323	H5
		Asp475	H12_	Tyr320	turn in H4			
		His449	H10/11	Lys367	H7			
		Met364	H6_H7	Lys367	H7			
		Arg 397	H8_H9	Glu324	H5			
2GTK	208	Glu460	H10/11_H12	Arg357	H6_H7	F1	Tyr473	H12
		Arg357	H6_H7	Glu276	H2'_H3	F1	His449	H10/11
		His466	H10/11_H12	Gln286	H3	F2	His323	H5
		Ile472	H12	Lys319	H4	F2	Ser289	H3
		Lys474	H12_	Lys319	H4			
		His449	H10/11	Lys367	H7			
		Lys367	H7	Phe363	loop in H7			
		Arg397	H8_H9	Glu324	H5			
		Asp396	H8_H9	Arg443	H10/11			
1PRG chain A		Glu460	H10/11_H12	Arg357	H6_H7			
		Arg357	H6_H7	Glu276	H2'_H3			
		Leu468	H12	His466	H10/11_H12			
		Asp475	H12_	Gln454	H10/11			
		Arg397	H8_H9	Glu324	H5			
		Asp396	H8_H9	Lys438	turn in H10/11			
		Met364	loop in H7	Lys367	H7			
		Lys367	H7	Phe363	loop in H7			
		Ser289	H3	Cys285	H3			
1PRG chain B		Glu471	H12	Lys474	H12_H7			
		His449	H10/11	Lys367				
		Arg397	H8_H9	Glu324	H5			
		Asp396	H8_H9	Lys438	H10/11			

AA, amino acid; SE, secondary structure the particular amino acid belongs to; PHF, pharmacophore feature; H2'_H3, structure between helices H2' and H3; H6_H7, structure between helices H6 and H7; H8_H9, structure between helices H8 and H9; H10/11_H12, structure between helices H10/11 and H12; H12_, structure after H12.
